# Multifocal squamous cell carcinoma symmetrically distributed on the feet: Diagnostic considerations

**DOI:** 10.1016/j.jdcr.2026.08.052

**Published:** 2026-09-06

**Authors:** Edwin Antonio, Camilla A. Cascardo, Michael J. Davis

**Affiliations:** a University of Washington School of Medicine, Seattle, Washington; b Department of Dermatology, University of Michigan, Ann Arbor, Michigan

**Keywords:** acitretin, acral, neuropathy, squamous cell carcinoma

## Introduction

Squamous cell carcinoma (SCC) commonly arises on chronically sun-exposed areas, such as the head, neck, and hands.^1^ Acral presentations, however, are rare and often mistaken for xerosis, calluses, non-healing wounds, or warts.^2^^,^^3^ Bilateral, symmetric lesions on the feet are even more uncommon and can be challenging to distinguish from hyperkeratotic inflammatory conditions. Here, we report a peculiar case of multifocal SCC primarily involving the lateral aspects of both feet in a patient with a long-standing history of peripheral neuropathy and diabetic foot ulcers.

## Case report

A woman in her 80s with a history of chronic diabetic neuropathy presented from an outside dermatologist for surgical evaluation of recalcitrant bilateral acral hyperkeratotic plaques. These lesions, which were initially diagnosed as calluses and non-healing ulcers, progressively worsened over 8 years, significantly impacting her quality of life due to pain and difficulty wearing shoes. Topical keratolytics, steroids, antibiotics, antifungals, intermittent paring, and cholesterol 2%/lovastatin 2% cream were trialed with no notable improvement. Additionally, lower extremity dopplers and pulse volume recordings at the time showed no significant peripheral arterial disease.

Prior to her presentation, the patient underwent shave biopsies of hyperkeratotic plaques on the left lateral foot and left lateral heel. Histopathology of the lateral heel plaque demonstrated squamous cell carcinoma in situ (SCCis) and was treated with electrodesiccation and curettage, while the lateral foot specimen showed an atypical well-differentiated squamoproliferative lesion with involved margins, prompting referral for further evaluation. Independent institutional review by our dermatopathologists of these outside biopsies supported the diagnosis of SCCis.

Upon examination of the feet, numerous symmetrically distributed hyperkeratotic papules and plaques were seen on an ill-defined erythematous base with notable sparing of the soles and mild hyperkeratosis in the periungual areas (Fig 1). Additionally, no scaling was present within the inter-webbed spaces of the toes, no nail changes were present, and the hands were completely spared. KOH scrapings and a mineral oil prep were negative for fungal elements and mites, respectively.

**Fig 1:**
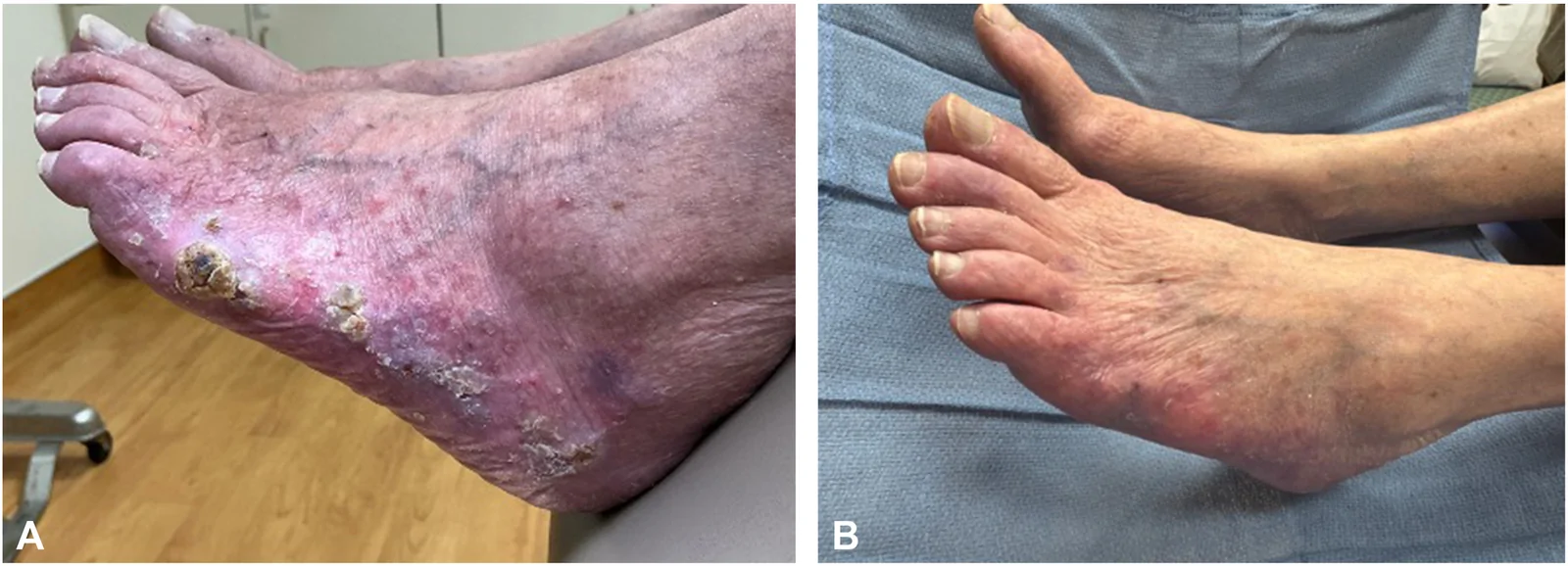
**A,** Left lateral foot demonstrating numerous ill-defined hyperkeratotic papules and plaques on an erythematous base with mild periungual hyperkeratosis. Comparable findings were present on the right lateral foot. **B,** Left lateral foot demonstrating significant improvement in previous hyperkeratotic papules and plaques. Comparable improvement occurred on the right lateral foot.

An extensive exposure history was taken, including exposure to arsenic, repetitive trauma, heat, heavy metals, ultra-violet, and frequent x-rays, which was largely unrevealing. Additionally, she denied any history of smoking, eczema, psoriasis, or plantar verruca. While likely not a significant exposure, the patient reported approximately 4 to 5 lifetime exposures to a shoe-fitting fluoroscope around the ages of 6 to 10-years-old.

Repeat in-house biopsies of keratotic and non-keratotic areas supported the prior diagnosis of SCC with full-thickness cytologic atypia and no evidence of an inflammatory process (Fig 2). A full review of systems was obtained and was negative for concerning symptoms. Her age-appropriate cancer screenings were up-to-date and unrevealing, as well as complete blood counts and metabolic chemistries. Furthermore, her case was presented at our institutional multidisciplinary department-wide conference for group consensus regarding the final diagnosis, and it was ultimately decided that her diagnosis was consistent with multifocal SCC.

**Fig 2:**
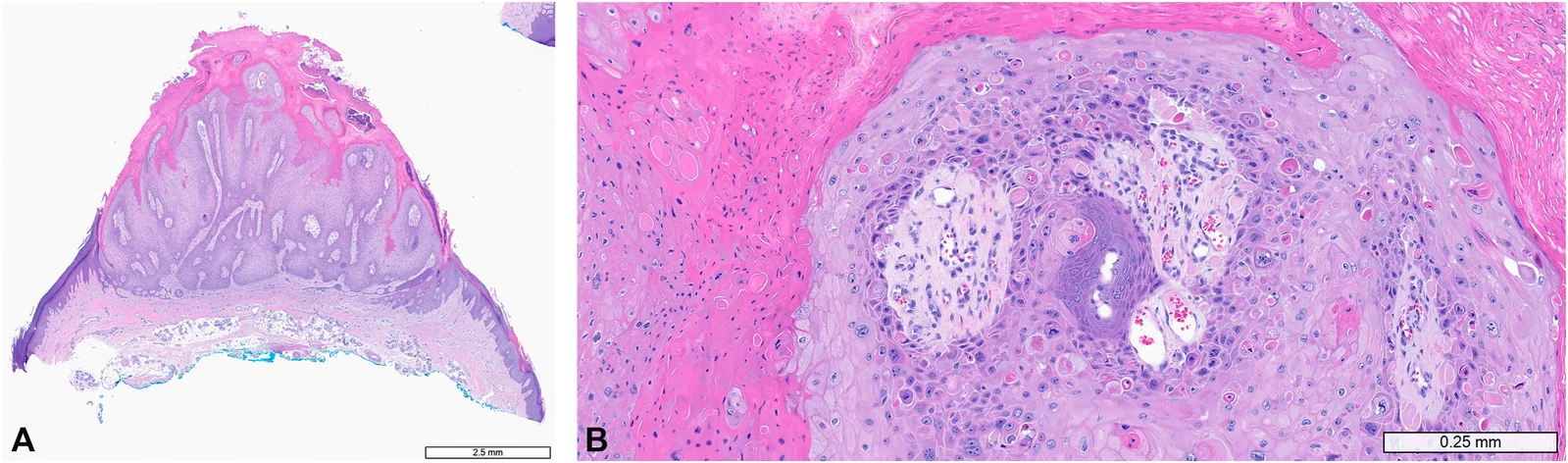
**A,** H&E 10× demonstrating an endophytic squamous proliferation with significant hyperkeratosis and pleomorphism without accompanying inflammation, consistent with a well-differentiated squamous cell carcinoma. **B,** H&E 40× demonstrating full-thickness cellular atypia and pleomorphism.

Treatment consisted of a multimodal approach. Oral acitretin 25 mg daily was initiated and subsequently reduced to every other day dosing after one month due to hypertriglyceridemia. This regimen was continued for 5 months before discontinuation due to alopecia. The two thickest and most concerning plaques were excised and allowed to heal by secondary intention, although intralesional 5-fluorouracil was considered. She completed 3.5 weeks of topical 5-fluorouracil before stopping due to an appropriately robust response. At each visit, keratotic lesions were pared down and treated with cryotherapy, and she otherwise used over-the-counter urea-based keratolytics throughout her treatment course. Near clearance was achieved within 6 months and the patient was followed for an additional 5 months with no clinical evidence of recurrence.

## Discussion

This case demonstrates a rare presentation of extensive bilateral SCC on the feet. The etiology of acral SCC is varied and often multifactorial, commonly arising in the setting of chronic trauma and/or inflammation.^2^^,^^4^ While not fully elucidated, the proposed mechanism for development of SCC in this location is insult-based transformation in the setting of preexisting chronic inflammatory conditions, such as lichen planus or eczematous dermatitis, HPV-related lesions, immunosuppression, and exposures to UV radiation or heavy metals.^4^^,^^5^

Several unique documented causes of acral SCC include arsenic exposure, radiation, ulcers, and paraneoplastic entities.^6^^,^^7^ While arsenic exposures in the United States are quite rare, various occupations and drinking water sources can put patients at risk with presentations occasionally occurring years after exposure. Fortunately, upon further questioning our patient had not worked with any chemicals or heavy metals, and did not grow up in a rural area, near manufacturing plants, or drinking well-water. Shoe-fitting fluoroscopes, also known as a “pedoscopes” were devices commonly used in the early 20^th^ century to determine shoe size, with reports demonstrating high variability in the amount of radiation exposure.^8^ While this was a potential exposure identified in our patient, her 4 to 5 lifetime exposures were fairly limited in comparison to prior reports of acral SCC development in the setting of greater than 100 lifetime exposures.^8^ Marjolin’s ulcers are also recognized causes of SCC on the lower extremities, which may result from long-standing diabetic or pressure ulcers.^2^

While our patient did have a history of diabetic ulcers on the feet, her development of SCC exceeded the distribution of prior ulcerations. Additionally, when SCC arises on acral surfaces without clear risk factors, clinicians should begin to consider paraneoplastic dermatoses, particularly acrokeratosis paraneoplastica also known as Bazex syndrome, presenting as psoriasiform plaques typically affecting the ears, nose, hands, and feet, along with onychodystrophy.^9^^,^^10^ Fortunately, our patient did not have any additional symptoms concerning for malignancy and was up-to-date on her age-appropriate cancer screenings.

In conclusion, bilateral, symmetric acral plaques that remain scaly, tender, and refractory to antifungal or anti-inflammatory therapy warrant early biopsy to exclude SCC. Acral SCC can closely mimic xerosis, tinea, verruca, or hypertrophic lichen planus, delaying recognition.^3^ This case underscores that recalcitrant acral hyperkeratotic plaques without clear etiology should prompt suspicion for malignancy even in the absence of known risk factors. Treatment depends on the extent of disease, but can require a combination of topical, surgical, and systemic approaches.

### Declaration of generative AI and AI-assisted technologies in the writing process

None.

## Conflicts of interest

None disclosed.
